# Immediate Postoperative Insulin Requirements May Predict Metabolic Outcome after Total Pancreatectomy and Islet Autotransplantation

**DOI:** 10.1155/2020/9282310

**Published:** 2020-12-21

**Authors:** Neha Verma, Amer Rajab, Jill Buss, Luis Lara, Kyle Porter, Philip Hart, Darwin Conwell, W. Kenneth Washburn, Sylvester Black, Kristin Kuntz, Shumei Meng

**Affiliations:** ^1^The Ohio State University Wexner Medical Center, Columbus, OH, USA 43210; ^2^Essen Medical Associates, Bronx, New York, NY, USA 10452; ^3^The Ohio State University, Columbus, OH, USA 43210

## Abstract

Chronic pancreatitis (CP) is a progressive disease that leads to eventual loss of endocrine and exocrine function. Total pancreatectomy and islet autotransplantation (TPIAT) is a treatment option for patients with CP; however, predicting postoperative metabolic outcomes remains elusive. In this single-center retrospective study, we report pre-TPIAT characteristics, beta cell function indices, islet yield, and post-TPIAT glucose management data to further understand their relationship. Islet yield, glucose level, and insulin requirement for 72 hours postoperatively were collected for a total of 13 TPIAT recipients between 9-2013 and 9-2018. In addition, their glucose control and basal insulin requirements at 3, 6, and 12 months post-TPIAT were analyzed. All 13 subjects had normal baseline fasting glucose levels. Median islet yield was 4882 IEq/kg (interquartile range 3412 to 8987). Median postoperative total insulin requirement on day 3 was 0.43 units/kg. Pre-TPIAT baseline glucose, insulin, or c-peptide level did not have a significant correlation with the islet yield. Similarly, there was no correlation between islet yield and insulin requirement at 72-hour postoperatively. However, there was an inverse correlation between the absolute islet yield (IEq) and insulin requirement at 6 months and 12 months following post-TPIAT. Further analysis of the relationship between 72-hour post-op insulin requirement and insulin requirement at discharge, 3, 6, and 12 months showed a positive correlation. Despite the finding of inverse correlation of islet yield with long-term basal insulin requirement, this study was not able to detect a correlation between the preoperative parameters to postoperative short-term or long-term outcome as noted in other studies. The 72-hour postoperative insulin requirement is a helpful postoperative predictor of patients needing long-term insulin management following TPIAT. This observation may identify a high-risk group of patients in need of more intensive diabetes education and insulin treatment prior to hospital discharge.

## 1. Introduction

Chronic pancreatitis (CP) is a progressive disease that is characterized by inflammation and fibrosis of the pancreas eventually leading to endocrine and exocrine dysfunction along with significant morbidity and mortality [1]. CP can be caused by multiple etiologies. The incidence of CP is estimated to range from 5 to 14 per 100,000 individuals with a prevalence of 30-50 per 100,000 individuals in most countries, while some countries have noted prevalence as high as 20-125/100,000 [1]. The incidence has been increasing over the past couple of decades based on various population studies worldwide [1].

CP is a debilitating disease; its morbidity and mortality is associated with eventual loss of endocrine function leading to secondary diabetes, along with gastrointestinal malabsorption due to loss of exocrine function as the disease progresses [2]. A reduction in work days is common due to repeated hospitalizations for excruciating abdominal pain and acute pancreatitis flares, and approximately 37% of CP patients face lower income after the diagnosis [1]. Approximately 90% of patients with CP suffer from refractory pain with a 20-25-year mortality of approximately 50% [2, 3].

Many treatment approaches have been utilized for CP patients including endoscopic procedures for obstructive disease process, surgery, and substitute therapy with insulin and pancreatic enzyme along with analgesics. However, since the mid-twentieth century, total pancreatectomy has become the treatment of choice for pain control for end stage CP [4, 5]. Studies have shown significant improvement in pain control with total pancreatic resection; however, it leaves patients with lifelong difficult to control diabetes due to loss of not only the beta cells but also various counterregulatory hormones. In order to combat the drawbacks of total pancreatectomy, there is significant merit in replacing autologous islet cells at the time of surgery for chronic pancreatitis. The very first Total Pancreatectomy with Islet Autologous Transplant (TPIAT) was carried out in 1977 at the University of Minnesota [6]. Since then, over the past 4 decades, there have been significant advancements in isolation and purification of the islet cells which has led to improved but inconsistent outcomes in terms of postoperative endocrine islet cell function recovery and pain resolution [6]. This is due to many factors such as preoperative patient factors as well as postoperative management targets that are not yet well understood. There have been many studies conducted to understand the ideal timing of TPIAT in the disease course, ideal preoperative pancreatic islet cell reserve, and postoperative glucose control to improve outcomes. Higher islet cell yield has been suggested as a positive predictor of insulin dependence postoperatively in some studies [7, 8]. Studies report variable islet yield as “high,” ranging from 2000 islet equivalent (IEq) per kilogram (kg) to 7000 IEq/kg [8, 9]. There are many baseline measurements such as the oral glucose tolerance test (OGTT), fasting blood glucose, and c-peptide that are assessed preoperatively to estimate patient's insulin production [7, 8, 10]. In this study, we report pre-TPIAT characteristics, beta cell function indices, and post-TPIAT glucose management from a single academic medical center to further understand the factors that may affect patient metabolic outcomes.

## 2. Material and Methods

### 2.1. Patient Selection and Data Collection

Potential candidates for TPIAT were selected from a pool of patients with CP at The Ohio State University Wexner Medical Center. These patients were evaluated by a multidisciplinary team that included gastroenterologists, transplant surgeons, an endocrinologist, a psychologist, and a nutritionist. After a comprehensive review of their clinical history, prior interventions and current quality of life, pain control, and metabolic status, it was determined if they would benefit from TPIAT. The potential TPIAT candidates then underwent further assessment of beta cell function via intravenous glucose tolerance test (IVGTT) and an oral mixed meal tolerance test (OMMTT) along with indices for beta cell function such as c-peptide levels [10]. For the OMMTT, the patient consumed “Boost high protein” drink over 5 minutes after an 8-hour overnight fast. Serum samples for glucose, insulin, and c-peptide were collected at baseline, 30, 60, 90, and 120 minutes after the drink consumption. Similarly for the IVGTT, patients were instructed to fast overnight for at least 8 hours prior to the test. Patients were also instructed to skip their evening and morning insulin along with any antihyperglycemic medications. For the test, one ampule of D50 was administered via the intravenous (IV) line; samples of c-peptide and serum glucose were obtained every 5 minutes for a total of 35 minutes. The first blood draw for this test is 5 minutes prior to the D50 infusion.

All selected candidates for TPIAT underwent preoperative counseling regarding the nature of the surgery, the potential risks and the benefits of TPIAT, and the potential risk for secondary Diabetes Mellitus (DM) due to TPIAT and post-operative DM management. Please refer to the following paper for detailed methods of the TPIAT at the Ohio State University Wexner Medical Center [11].

On the day of TPIAT, glucose was monitored frequently, and immediately postoperatively intravenous insulin was initiated to maintain near euglycemic control. Immediately, postoperatively, insulin infusion protocol was initiated with target blood glucose range of 100-120 mg/dL. Glucose was checked and recorded every hour for the first 48 hours. Thereafter, if glucose remained in target range of 100-120 mg/dL consistently for four hours, then glucose monitoring was relaxed to every 2 hours. Postoperatively, patients were NPO and maintained on IV fluids containing D5W at 50 cc/hour. Along with the islet yield, glucose levels, as well as insulin requirements, were monitored closely and recorded for 72 hours postoperatively. In addition, patient's glucose control information was also collected at 3 months, 6 months, and at one year postoperatively via their HbA1c and basal insulin use.

All data were collected according to approved Institutional Review Board (IRB) protocol (#2018H0429). As the study was retrospective, with chart review involving the use of existing data, and there was minimal or no risk to participants, it had an IRB and patient consent “exempt” status under regulations concerning human subjects.

### 2.2. Statistics

Patient characteristics were summarized as median and interquartile range. The correlation between islet yield and (1) baseline values (glucose, insulin, and c-peptide) and (2) insulin requirement (3-day postoperative and long-term follow-up) was analyzed by calculation of Pearson correlation coefficients. The correlation between 3-day postoperative insulin requirement and long-term insulin requirement were also analyzed by calculation of Pearson correlation coefficients. Assumptions of normality and absence of outliers were assessed visually through bivariate scatterplots. Analyses were performed in SAS software version 9.4 (SAS Institute, Cary NC, USA).

## 3. Results

We identified a total of 13 patients that met the criteria to undergo TPIAT between 9-2013 and 9-2018. Their median age was 45 with an interquartile range (IQR) of 24 to 52 years. Among them, 61.5% were women, which is consistent with overall female dominant prevalence of chronic pancreatitis. All patients were Caucasian. Median duration of pancreatitis was 5 years and the cause of pancreatitis was mainly idiopathic with the rest being secondary to genetic mutations. All 13 patients had a baseline fasting glucose in range (min-max) of 60-125 mg/dL with a very large variation of baseline insulin level (IQR 0.5-10.4 uIU/mL) and more consistent baseline c-peptide (IQR 1.15-2.15 ng/mL). The majority of those patients had acceptable islet yield with IQR of 3412 to 8987 with median of 4882 IEq/kg.

We also collected the 3-day postoperative total insulin requirement via the insulin ggt, while the patients were NPO and on maintenance IV fluid of D5W at 50 cc/hour. The 3-day postoperative total insulin requirement was approximately 0.43 units/kg with an interquartile range of 0.29 to 0.71 units/kg. Thereafter, basal insulin requirements were collected at discharge and long-term follow-up at 3, 6, and 12 months (Table 1). Due to variable po intake and corresponding meal covering insulin regimens, only basal insulin information was able to be accurately recorded. Therefore, we report basal insulin doses for each patient at 3, 6, and 12 months follow-up to reflect their steady insulin requirement.

Five of the 13 patients were lost to follow-up at the 12 month timepoint post operatively. Out of the remaining 8 patients, 6 patients had an HbA1c < 6.5% at the 12-month postoperative follow-up. Only 2 of the 6 patients were on 4.75 and 6 units of basal insulin, while other 4 patients were insulin independent. Two out of the total eight patients had an HbA1c > 6.5% and were on basal insulin of 12.8 units to 14.4 units at the 12 mo follow-up. Of note, both patients with HbA1c > 6.5% had an islet yield < 3500 IEq/kg, whereas patients with well controlled diabetes (HbA1c < 6.5%) had an islet yield > 3500 IEq/kg.

The association between islet yield (IEq) and baseline values (glucose, insulin, and c-peptide), insulin requirement at 3-day postoperative, and long-term follow-up were analyzed. Pearson correlation coefficients were calculated to assess the correlation between the measures listed previously.

**Figure 1:**
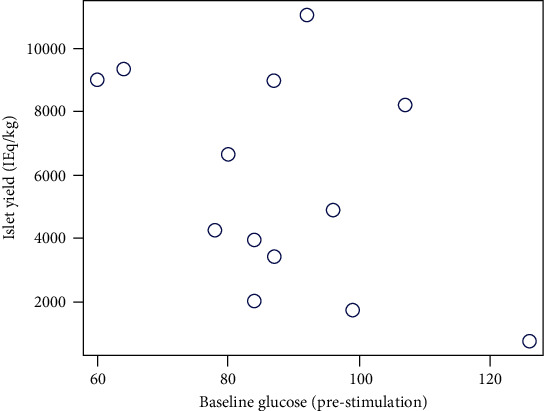
Preoperative baseline glucose prestimulation with glucose plotted against islet yield per kilogram (IEq/kg) intraoperatively. The analysis revealed no statistically significant correlation of islet yield (IEq/kg) with preoperative baseline glucose.

**Figure 2:**
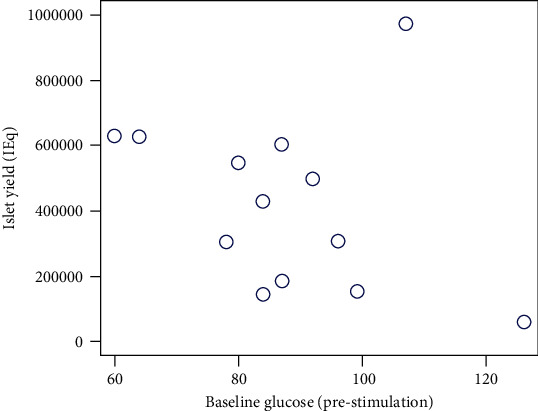
Preoperative baseline glucose prestimulation with glucose plotted against actual islet yield (IEq) intraoperatively. The analysis revealed no statistically significant correlation of absolute islet yield (IEq) with preoperative baseline glucose.

The analysis revealed no statistically significant correlation of islet yield with preoperative baseline glucose (Figures 1 and 2), insulin level, or c-peptide levels. Similarly, there was no direct correlation noted between islet yield and the 3-day insulin requirement postoperatively. However, there was an inverse correlation noted between the islet yield in total IEq and insulin requirement at 6 months and 12 months post-TPIAT follow-up. Similar correlation was seen between islet yield in IEq/kg and insulin requirement at postsurgery 6 months, but the correlation between islet yield in IEq/kg and insulin requirement at 12 months post-surgery only showed a similar trend with a *p* value of 0.06 (Table 2).

**Figure 3:**
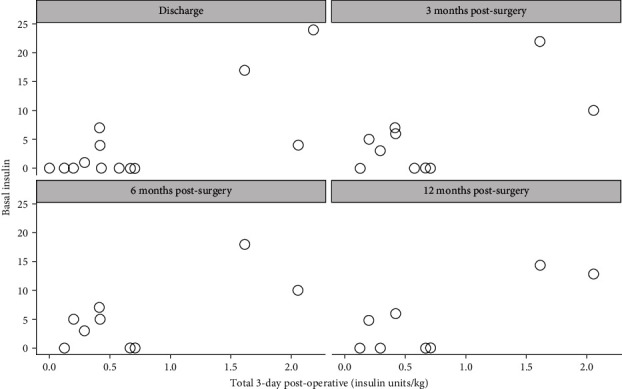
Postoperative basal insulin requirement per kilogram (insulin units/kg) in the immediate post-operative period of 72 hours showed a positive correlation with basal insulin requirement (units) at 3 months, 6 months, and 12 months postoperatively. These correlations were consistently strong and significant through 12 months postsurgery.

**Figure 4:**
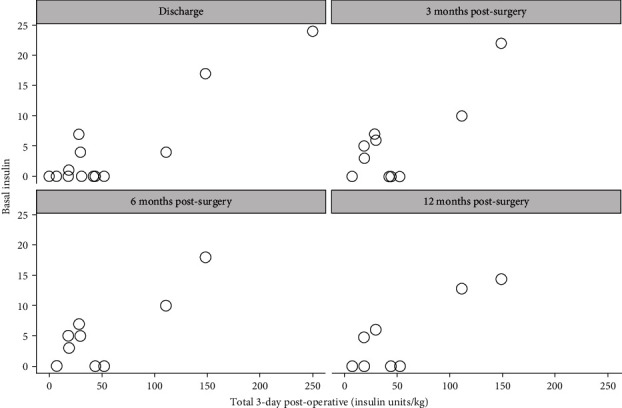
Absolute postoperative basal insulin requirement per kilogram (insulin units/kg) in the immediate postoperative period of 72 hours showed a positive correlation with basal insulin requirement (insulin units) at 3 months, 6 months, and 12 months postoperatively. These correlations were consistently strong and significant through 12 months postsurgery.

Further analysis of the relationship between immediate postop insulin requirement over 3 days and insulin requirement at discharge, 3 months, 6 months, and 12 months showed a positive correlation. These correlations were consistently strong and significant through 12 months postsurgery (Table 3, Figures 3 and 4).

## 4. Discussion

From this single-center cohort of TPIAT patients, we demonstrated that CP caused by various etiologies can benefit from TPIAT with glucose control in addition to their pain control. Careful assessment with two different tests (IVGTT and OMMTT) can help select the candidates for potential successful TPIAT procedure as well as clear metabolic benefit.

While CP in children is most commonly caused by genetic factors such as cationic trypsinogen gene (PRSS1), serine protease inhibitor Kazal type 1, cystic fibrosis transmembrane conductance regulator (CFTR), and chymotrypsin C (CTRC) mutations, most of the patients in this study, who were all adults, had idiopathic CP. This makes the timing of the TPIAT critical with the right presurgical evaluation to select the ideal candidate [12]. Previous studies used the fasting glucose from OGTT or an absolute c-peptide level from the OMMT to correlate with islet yield, for predicting long-term positive metabolic outcomes [8]. The research team at Johns Hopkins University was able to show a correlation between normal fasting glucose and OGTT pre-TPAIT with long-term insulin-free rate after TPIAT [7]. Other studies have failed to confirm this correlation. Many researchers are actively searching for better predictors. Recently, BETA-2 scores, a parameter computed from fasting data, HbA1c, and insulin requirement, have been proposed to not only better estimate islet function pre- and post-TPAIT but also for a long-term indicator of islet graft function following TPIAT [13, 14]. However, this needs to be further confirmed with larger studies.

Many studies used either IVGTT or OMMTT for patient selection for TPIAT to predict insulin independence post-TPIAT; however, the data is mixed for each test [8, 13]. Our study incorporated both previously published IVGTT and OMMTT to establish baseline characteristics [8]. Through our selection process, parameters from the IVGTT and the OMMTT were collected. Their relationship with islet yield was analyzed. Our data failed to demonstrate any direct correlation between our IVGTT or OMMTT parameter and baseline glucose, insulin level, or c-peptide value to islet yield. This could be due to the small number of patients in the study; moreover, not every patient was able to tolerate the OGTT, and some had difficulty with IVGTT. Therefore, we were not able to obtain data for both tests for every patient for an accurate analysis. These tests are done preoperatively to better select candidates who are more likely to have success post-TPIAT. Certainly, larger studies are needed to fully understand the true potential of these tests when used alone or together.

Consistent with previous reports [8, 10, 15] our results confirmed that islet yield inversely correlates with the long-term insulin requirement, indicating that the islet yield is a significant determining factor that affects the outcome of glucose metabolism after TPIAT.

For the first time, our study showed a positive correlation between postoperative basal insulin requirement for 72 hours and the long-term basal insulin requirement at 3, 6, and 12 months. Due to the difficulty of recording accurate prandial insulin doses that is affected by fluctuation of carbohydrate intake, we used the basal insulin doses at 3, 6, and 12 months to better reflect their islet functionality. It has been noted that all our 13 study patients were kept NPO and were given standard maintenance IV fluids without eternal TF or TPN for 3 days postoperatively, and their total insulin requirements during that time equal to their basal insulin requirements. This positive correlation between immediate postoperative basal insulin requirements and long-term postoperative basal insulin need could be very useful in predicting long-term metabolic outcome of TPIAT patients. This predictive value can be used to identify patients that can be targeted with more diabetes education and better insulin regimen for their glucose management at discharge.

This study showed a correlation between islet yield and long-term basal insulin requirement and a correlation between postoperative basal insulin requirement and long-term basal insulin need. The islet yield did not significantly correlate to the insulin requirement in the immediate 72-hour postoperative period. This could be due to the small sample size; however, other possible explanations need to be investigated. With further addition of patients to our cohort in the future, the answer to this question can be further addressed.

Our study has several limitations; it is a retrospective observational study. As such, the sample size is small with only 13 patients, this can cause low detection power especially when we failed to demonstrate any direct correlation between IVGTT and OMMTT parameters to short-time or long-term metabolic outcomes. It is well known that patients with chronic pancreatitis are a unique group of patients having high noncompliance rate. Patient lost to follow-up is not an uncommon thing despite the tight selection criteria. The missing data could affect our analysis adversely especially with findings of negative correlations. We hope with bigger data series in the future; this limitation can be better overcome.

CP is a debilitating disease that can be effectively treated with TPIAT if patients are carefully selected. TPIAT can provide significant metabolic benefit in addition to obvious pain relief and improved quality of life. Our TPIAT patients underwent rigorous multidisciplinary evaluation and discussion, which could explain the positive outcome for the patients requiring minimal insulin post-TPIAT. It brought up a debate about whether we should include borderline DM patients for TPIAT to provide partial metabolic benefit given the well-accepted benefit of pain control and improvement of quality of life.

The long-term follow-up for the TPIAT patients is the strength of our study which helped us understand their metabolic outcomes not just immediately postop but also long-term post-TPIAT. In conclusion, to our knowledge, this study is the first to demonstrate that immediate postoperative basal insulin requirement after TPIAT is a direct predictor for long-term metabolic outcome. This can help identify the patients that need aggressive DM education and close follow-up regarding their glucose management.

## Figures and Tables

**Table 1:** Patient Characteristics, *n* = 13.

Variable	Median (IQR) or *n* (%)	*n* missing
Age	45 (24-52)	0
Sex, female	8 (61.5%)	0
Race, Caucasian	13 (100%)	0
Duration of pancreatitis, years	5 (4-10)	0
Diagnosis/etiology of pancreatitis		0
Idiopathic CP	6 (46.2%)	
Idiopathic RAP	3 (23.1%)	
Hereditary (PRSS1) CP	3 (23.1%)	
CFTR mutation, CP	1 (7.7%)	
Baseline glucose (pre-stim)	87 (80-96)	0
Baseline insulin (pre-stim)	7.6 (0.5-10.4)	2
Baseline c-peptide (pre-stim)	1.70 (1.15-2.15)	3
Islet yield		
IEq (1000 s)	427.6 (185.6-604.8)	0
IEq/kg	4882 (3412-8987)	0
3-day postoperative basal insulin		
Units/kg	0.43 (0.29-0.71)	0
Total units	30.4 (18.5-52.1)	0
Basal insulin units		
Discharge	0 (0-4)	0
3 months postsurgery	4 (0-7)	3
6 months postsurgery	5 (0-7)	4
12 months postsurgery	2.4 (0-9.4)	5

IQR: interquartile range.

**Table 2:** Correlations with islet yield. Pearson correlation coefficients (*r*, *p* value, *n*).

Measure	Islet yield (IEq)	Islet yield (IEq/kg)	*n*
Baseline glucose (pre-stim)	-0.31, *p* = 0.31	-0.46, *p* = 0.11	13
Baseline insulin (pre-stim)	-0.27, *p* = 0.43	-0.43, *p* = 0.19	11
Baseline c-peptide(pre-stim)	-0.33, *p* = 0.42	-0.47, *p* = 0.24	8
3-day postoperative basal insulin			
Total units	0.34, *p* = 0.25	-0.05, *p* = 0.87	13
Units/kg	0.15, *p* = 0.63	-0.14, *p* = 0.65	13
Basal insulin units			
Discharge	0.28, *p* = 0.36	-0.10, *p* = 0.75	13
3 months postsurgery	-0.44, *p* = 0.20	-0.47, *p* = 0.17	10
6 months postsurgery	-0.74, *p* = 0.02	-0.74, *p* = 0.02	9
12 months postsurgery	-0.71, *p* = 0.05	-0.69, *p* = 0.06	8

IEq: islet equivalent.

**Table 3:** Pearson correlation coefficients of 3-day basal insulin with basal insulin requirements at discharge, 3, 6, and 12 months.

3-day post-operative measure	Basal insulin units			
	Discharge *n* = 13	3 months *n* = 10	6 months *n* = 9	12 months *n* = 8
Total insulin, units/kg	0.76, *p* = 0.003	0.66, *p* = 0.04	0.69, *p* = 0.04	0.82, *p* = 0.01
Total insulin, units	0.91, *p* < 0.001	0.81, *p* = 0.004	0.82, *p* = 0.01	0.85, *p* = 0.01

## Data Availability

The data that support the findings of this study are available from the corresponding author, upon reasonable request.

## Additional Points

*Statement of Human and Animal Rights*. As the study was retrospective, with chart review involving the use of existing data, and there was minimal or no risk to participants.
